# *Clostridium septicum* Sepsis and Colon Carcinoma: Report of 4 Cases

**DOI:** 10.1155/2011/248453

**Published:** 2011-06-26

**Authors:** Eric Mao, Aine Clements, Edward Feller

**Affiliations:** ^1^The Warren Alpert Medical School of Brown University, Providence, RI 02912, USA; ^2^Division of Gynecologic Oncology, University of Iowa Hospitals and Clinics, Iowa City, IA 52242, USA

## Abstract

An association exists between colon carcinoma and *Clostridium septicum* infection, especially bacteremia. We reviewed retrospectively all positive blood cultures for this organism at a 300-bed general hospital over 4 years. Four of 15 cases were associated with concurrent colon carcinoma. *C. septicum* infection was the presenting feature of previously undiagnosed large bowel malignancy in three patients. We report this small case series to alert clinicians to the diverse spectrum and diagnostic difficulties of this rare, potentially catastrophic association. Although commonly associated with necrotizing skin or soft tissue infections, this bacterium can present with nonspecific or atypical symptoms. All patients with positive blood cultures for *C. septicum*, even without clinical suspicion of large bowel malignancy, should undergo colonoscopy to evaluate for colon carcinoma.

## 1. Introduction

There is a connection between certain bacterial infections and malignancy. The best-known relationship is between *Streptococcus bovis* infection and colon carcinoma, but there is another link between *Clostridium septicum* and large bowel malignancies [1–6]. Additionally, *C. septicum* has been associated with hematological malignancies, immunosuppression, neutropenia, and diabetes mellitus [2, 3]. We report 4 cases of *C. septicum* septicemia associated with large bowel malignancy to alert clinicians to the diverse, potentially misleading features of this life-threatening association.

## 2. Case Presentation

A retrospective review of blood culture results for *C. septicum* was performed over a 4-year period in a 300-bed general medical surgical hospital in Providence, RI. All patients with bacteremia caused by this organism were identified. Hospital charts were then assessed for any association with large bowel malignancy.

Of 15 patients with positive blood cultures for *C. septicum*, 4 had biopsy-documented colon carcinoma. The age range was from 73 to 81, including 3 men and 1 woman. Each patient had fever of 1–7 days duration. One patient had a history of colon cancer resected surgically in the prior year. Evaluation of this patient documented unsuspected local recurrence of cancer in the colon. In the remaining 3 cases, symptoms due to clostridial infection were the presenting features of previously undiagnosed cancer. Only 2 of 4 had symptoms or signs suggesting abdominal pathology. The tumor was located in the cecum in each case. One patient had bacterial aortitis. No patient had gas gangrene, a necrotizing skin, or soft tissue infection (Table 1). No patient had another underlying disorder reported to be associated with *C septicum* infection.

## 3. Discussion

Unlike *S. bovis*, *C. septicum* is not part of the normal bowel flora and is a rare cause of infection, accounting for as few as 1.3% of all blood cultures positive for clostridium [7]. Our results support previous evidence that *C. septicum* infections are associated with gastrointestinal malignancy. In a review of 320 cases dating back to 1969, 39% had a gastrointestinal malignancy [7]. Another review of 32 cases found that one-half of patients had concurrent colorectal cancer [8]. Another review of 163 cases found that 34% had an associated colon carcinoma [9]. The mortality rate of *C. septicum* sepsis is prohibitive, reported to range from 45% to 70% [2–4]. Virulence may be related to production of multiple toxins, aggressive tissue invasion, and infection in compromised hosts [10].

*C. septicum* is a gram positive, anaerobic, spore-forming rod which grows normally in soil. The postulated mechanism of infection in colon cancer involves disruption of the normal mucosal barrier due to tumor-induced ulceration, followed by bloodstream invasion. Anaerobic glycolysis in the tumor may provide an acidic and hypoxic environment facilitating spore germination [2]. Cecal tumors are most common, as in all of our patients, possibly due to pH, osmotic, and electrolyte characteristics conducive to growth of the organism [11]. Subsequently, the organism sporulates and spreads to local intestinal mucosa. With colonic mucosal disruption, *C. septicum* can spread hematogenously. Mucosal disruption can be caused by tumor necrosis, bowel perforation, surgery, radiation, or a medical procedure such as colonoscopy or barium enema [1, 2, 5, 6]. Impaired host immunity from alcohol abuse, steroids, atherosclerosis, diabetes, or neutropenia is also believed to facilitate translocation. 

The clinical spectrum of *C. septicum* is diverse (Table 2), most commonly presenting as cellulitis, fasciitis, myonecrosis, gas gangrene, or visceral or soft tissue abscess. A potentially catastrophic soft tissue manifestation is nontraumatic spontaneous gas gangrene. This organism, more aerotolerant than* C. perfringens*, is thus more likely to infect healthy tissue. Nonspecific symptoms of fever or abdominal pain are common [3].

Aortitis, a rare manifestation occurring in one of our patients, can present with nonspecific symptoms such as fever or abdominal pain. Diagnosis is most commonly made by CT scan showing a soft tissue prominence surrounding a normal aorta initially and later development of peri-aortic gas. It may also present as life-threatening aortic aneurysmal rupture. In a review of* C. septicum *aortitis, 21 of 23 cases were associated with colonic adenocarcinoma or polyps [11]. This infection has been proposed to result from hematogenous seeding of an atheromatous aorta from a distant source of bacteremia [6, 11]. A rare mechanism is direct extension of infection, such as from contiguous colonic perforation [11]. 

The diagnosis of *C. septicum*-associated large bowel malignancy may be delayed or missed. Clinical manifestations are commonly nonspecific, mimicking more common disorders. At times, no clinical clue to a colon malignancy is present. Some clinicians may be unaware of the association. Bacterial sepsis may be the initial feature of previously undiagnosed and unsuspected large bowel carcinoma.

 In our small series, 4 of 15 cases of *C. septicum* sepsis had associated large bowel malignancy. All patients with blood cultures positive for this organism should undergo colonoscopy, as in our 4 patients. Unexplained fever in patients with known large bowel cancer should prompt consideration of this organism. Because malignant tumors can occur as long as several years after infection, follow-up evaluation is imperative [5]. Although commonly associated with necrotizing skin or soft tissue infections, *C. septicum* can present with nonspecific symptoms or a variety of other infections and should prompt consideration of malignancy.

## Figures and Tables

**Table 1:** Cases of *C. septicum* infection.

Age	Gender	Hx of colon CA	Clinical presentation	Evaluation	Outcome
81	Male	Resected proximal colon CA	2 days of fever	CT: liver metastases Colonoscopy: recurrent carcinoma	Palliative care
87	Female	None	Steady, mild RLQ pain for 1 week and fever for 1 day	CT: cecal mass, liver metastases Colonoscopy: carcinoma	Palliative care
73	Male	None	Fever for 1 day, weakness unsteady gait	CT: thickened ascending colon wall; periaortic gas (aortitis) Colonoscopy: carcinoma	In-hospital death
76	Male	None	Fever with diffuse lower abdominal pain	CT: cecal mass Colonoscopy: carcinoma	Surgical resection

**Table 2:** Clinical spectrum of *C. septicum. *

Cellulitis	Septic Arthritis
Fasciitis	Septic shock
Myonecrosis (gas gangrene)	Abdominal pain
Abscess (visceral or soft tissue)	Fever, malaise
Aortitis	Hemolysis
Aortic aneurysm (ruptured or unruptured)	
